# Structure-Based Pipeline for Plant Enzymes: Pilot Study Identifying Novel Ginsenoside Biosynthetic UGTs

**DOI:** 10.3390/biotech14030073

**Published:** 2025-09-12

**Authors:** Kisook Jung, Ick-hyun Jo, Bae Young Choi, Jaewook Kim

**Affiliations:** 1 Department of Biology Education, Korea National University of Education, Cheongju 28173, Republic of Korea; sena0109@knue.ac.kr; 2 Department of Crop Science and Biotechnology, Dankook University, Cheonan 31116, Republic of Korea; 3 School of Liberal Arts and Sciences, Korea National University of Transportation, Chungju 27469, Republic of Korea

**Keywords:** protein 3D structure, enzyme activity, ginsenoside, UGT

## Abstract

Models that predict the 3D structure of proteins enable us to easily analyze the structure of unknown proteins. Though many of these models have been found to be accurate, their application in plant proteins is not always entirely accurate. Thus, we aimed to develop a versatile yet simple pipeline that can predict novel proteins with a specific function. As an example, via benchmark studies, we sought to discover novel UDP-glycosyltransferases (UGTs) potentially involved in ginsenoside biosynthesis. Since the functionality of these UGTs has been shown to be determined by a few amino acids, a 3D-structure-based pipeline was required. Our pipeline includes four sequential steps: a sequence-based homology search, AlphaFold3-based 3D structure prediction, docking simulations with ginsenoside intermediates using SwissDock and CB-Dock2, and MPEK analysis to assess interaction stability. Through the application of this benchmark, we optimized the role of each module in the pipeline and successfully identified four novel UGT candidates. These candidates are predicted to catalyze the conversion of protopanaxadiol (PPD) to compound K (CK) or protopanaxatriol (PPT) to ginsenoside F1. This pilot study demonstrates how our pipeline can be used for the functional annotation of plant proteins and the discovery of enzymes involved in specialized pathways.

## 1. Introduction

Recent advances in artificial intelligence (AI) have enabled biologists to predict the three-dimensional (3D) structures of proteins with remarkable accuracy [1,2,3]. Since the function of proteins is fundamentally determined by their 3D structure, these predictive tools have opened up new avenues for aiding our detailed understanding of protein functionality. The development of such models has been greatly facilitated by the large number of experimentally resolved structures deposited in the Protein Data Bank (PDB) [4]. As of 5 May 2025, the PDB contained 130,492 eukaryotic protein structures. Of these, more than 97,000 entries correspond to proteins from the Animalia kingdom, while only 3990 structures represent plant proteins. This disparity highlights a significant limitation for plant biologists, as AI-based structural models remain undertrained when it comes to plant-specific protein datasets, limiting their reliability in plant science applications.

The function of a protein is largely determined by its structure, a concept that has inspired efforts to artificially design proteins with desired functions [5]. The advent of accurate energy-based modeling has enabled the rational design of proteins by allowing for the prediction of protein–ligand binding sites based on thermodynamic properties. The current prediction approaches cover two key aspects: (1) the affinity and probability of binding between a target protein and its ligand and (2) the physicochemical nature of the receptor–ligand interaction, both of which are addressed by a variety of dedicated computational tools [6,7,8,9,10]. Recent advances have also made it possible to estimate enzymatic properties such as catalytic constants and turnover rates [11,12]. Taken together, these tools provide a powerful framework for predicting the functionality of uncharacterized proteins that has the potential to be highly biologically significant.

Ginsenosides are triterpenoid saponins found primarily in *Panax ginseng*, and they are widely considered to be the major bioactive compounds responsible for the pharmacological effects of ginseng [13]. These compounds exhibit considerable chemical diversity, which translates into a variety of biological effects [13]. The composition and abundance of ginsenosides vary according to tissue type, plant age, and cultivation conditions [14,15,16,17,18]. The biosynthesis of ginsenoside involves a complex network of enzymes, including UDP-glycosyltransferases (UGTs) [19], which catalyze the transfer of sugar moieties to aglycones and thus play a critical role in determining the final structure and properties of each ginsenoside [19,20].

Among these enzymes, UGTs are of particular interest due to the role they play in defining the chemical diversity and activity of ginsenosides. To date, several UGTs involved in ginsenoside biosynthesis have been identified using conventional biochemical and molecular biological techniques [21,22,23]. However, a major bottleneck in the mass production of pharmaceutically valuable ginsenosides is the enzymatic conversion of protopanaxadiol (PPD) or protopanaxatriol (PPT) to compound K (CK) or ginsenoside F1, respectively. The discovery of novel UGTs responsible for these conversions has been slow, mainly due to the high sequence similarity between functionally active and inactive UGTs, which often differ by only a few amino acids [23]. To overcome this limitation, we developed a de novo computational pipeline, optimized for plant proteins, to accurately predict and prioritize candidate UGTs involved in ginsenoside biosynthesis. Using this pipeline, we identified four previously uncharacterized UGTs with structural and functional features suggestive of them playing a potential role in catalyzing the key steps in ginsenoside biosynthesis. These candidates require further experimental validation to confirm their enzymatic activity and functional relevance.

## 2. Materials and Methods

### 2.1. Identification of Sequence-Based Homolog Proteins

In previous research, we obtained the public accession of the target UGTs [21,23]. Using BLASTp 2.12.0+, where our criteria were an E-value of $1\times 10^{-5}$ and similarity of 99.9%, we identified the close homologs of the target UGTs [24,25]. Multiple sequence alignments of homologs were performed using MAFFT v7.490, followed by alignment trimming using trimAl v1.5.rev0 and maximum likelihood phylogenetic tree construction using IQ-TREE2 [26,27,28]. To investigate the conservation of the Plant Secondary Product Glycosyltransferase (PSPG) motif among UGT homologs, sequences were aligned using the MUSCLE algorithm implemented in MEGA X v11.0.13, and these were visualized accordingly [29,30].

### 2.2. Prediction of 3D Protein Structures and Observation

To assess the accuracy of structure prediction models for plant UGTs, both AlphaFold2 Version 2.0 and AlphaFold3 (https://alphafoldserver.com/) (3 September 2025) were used to generate structural models for three experimentally resolved plant UGTs [1,2]. The predicted structures were compared with experimentally determined structures using pairwise structural alignment to calculate the root mean square deviation (RMSD) values [31]. SPSS software 21 was used for data visualization, and Student’s *t*-test was used to assess the differences in the statistical significance of RMSD [32]. Structural visualization and qualitative analysis of the predicted models were performed using ChimeraX 1.10.1 [33]. In addition, a structure-based phylogenetic tree was generated using the Dali server (http://ekhidna2.biocenter.helsinki.fi/dali/) (3 September 2025) to assess the structural relationships between UGTs [34].

### 2.3. Prediction of UGT–Ligand Interaction and Functionality

To predict the compatibility of candidate UGTs to bind with ligands, the ligand structures in SMILES or MOL2 format were retrieved from the PubChem database. The selected protein–ligand pairs were analyzed using the molecular docking tools SwissDock (https://www.swissdock.ch/) (3 September 2025) and CB-Dock2 (https://cadd.labshare.cn/cb-dock2/index.php) (3 September 2025), which assess binding affinity and interaction geometry [8,9,10]. To verify the stability of protein–ligand interactions, we performed molecular dynamics analysis with myPresto version 5, only accounting for the protein and ligand and default settings [35]. Functional parameters such as the catalytic turnover number (k_cat) and Michaelis constant (K_m) were estimated using the MPEK platform, which provides computational predictions of enzyme kinetics based on structural and energetic features [12].

### 2.4. Detailed Verification Through Molecular Dynamics Simulation

To verify our pipeline, we performed molecular dynamics analysis with GROMACS version 2021.4-Ubuntu-2021.4-2 as follows [36]. Open Babel 3.1.1 was applied to generate ligand composition with hydrogen atoms [37]. Then, AnteChamber PYthon Parser interfacE v. 2023.10.2 was used to make ligand topology file [38]. Protein and ligand topology were merged in a cubic topology with -c -d 0.6 -bt cubic options. Then solation was performed with the spc216 model. Ion condition was set as 150 mM of potassium and 5 mM of sodium, which mimics the typical plant cytoplasm. Energy minimization was performed with the following settings: integrator = steep; emtol = 1000.0; nsteps = 50,000; energygrps = System. Then, molecular dynamics analysis was performed for 150 ns, with a single step simulating 3 ps, and thus 50,000,000 steps were assessed. To identify the important residue in the interaction between UGTs and ligands, we performed bibliographic analysis and CLUSTALW2 to reveal the exact position in each protein [39,40].

## 3. Results

### 3.1. Identification of Close Homologs of Ginsenoside Biosynthetic UGTs

**Figure 1:**
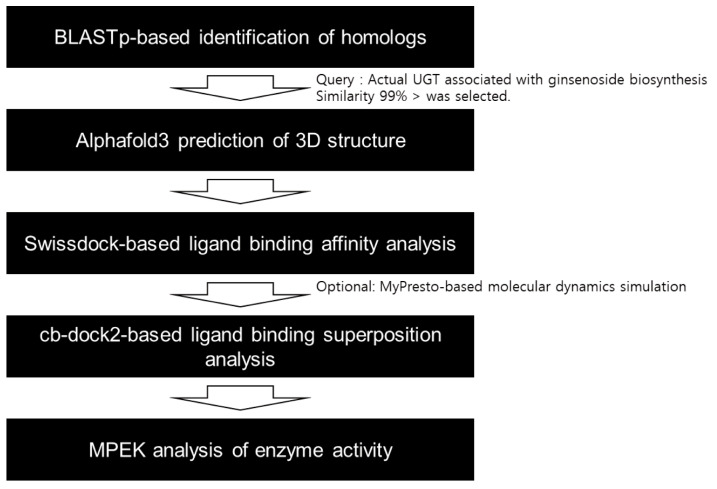
Schematic diagram depicting the 3D-structure-based strategy to identify the novel ginsenoside biosynthetic UGTs.

**Figure 2:**
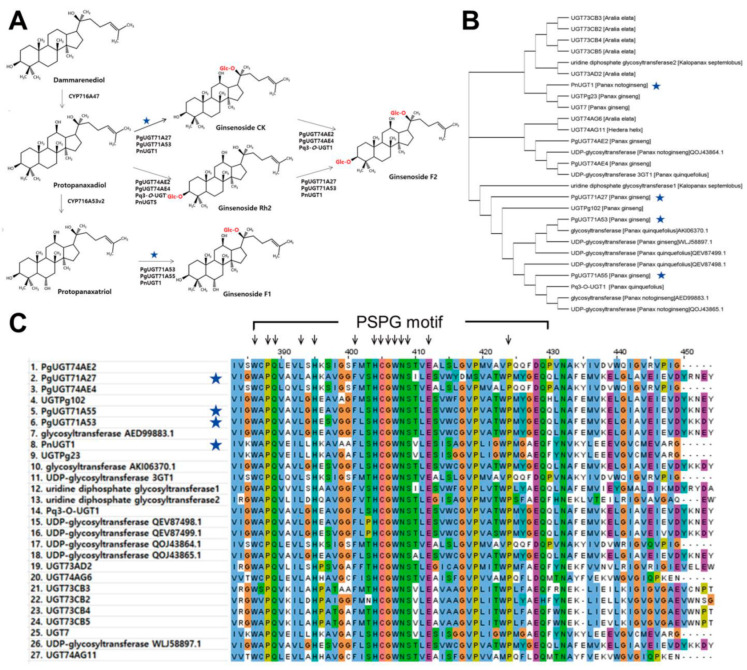
Identification of highly close homologs on the UGTs of interest in Table 1. Blue stars indicate the catalytic steps of interest in this study. (**A**) Schematic diagram depicting the ginsenoside biosynthetic process. (**B**) Phylogenetic tree based on the sequence similarity of 27 close homolog UGTs, including experimentally verified UGTs. Cladogram visualization was applied for visibility. (**C**) Sequence alignment near the PSPG motif of 27 close homolog UGTs. Arrows indicate the highly conserved residues in the PSPG motif. In detail, amino acids in position number 386, 388, 389, 393, 395, 401, 404, 405, 406, 407, 408, 409, 412, and 424 are shown to be associated with interacting UDP-sugar moieties and substrate specificity.

To identify novel UGTs potentially involved in ginsenoside biosynthesis, we developed a structure-based prediction pipeline (Figure 1). To demonstrate the utility of our pipeline, we focused on two critical enzymatic steps in ginsenoside biosynthesis: (1) the glycosylation of protopanaxadiol (PPD) to compound K (CK) and (2) the glycosylation of protopanaxatriol (PPT) to ginsenoside F1 (Figure 2; Table 1). To date, four UGTs have been experimentally confirmed to catalyze these reactions (Figure 2A; Table 1). Using these four functionally characterized UGTs as queries, a BLASTp search was performed against the NCBI nr database. Further, homologs were selected using stringent criteria (E-value ≤ 1 × 10^−5^ and ≥99.9% identity), resulting in the identification of 23 candidate UGTs from different species (Figure 2B). Although not every clade contained a direct homolog of functionally validated UGTs, each clade included at least one of the known UGTs (Figure 2B). To further confirm the validity of the selected candidates, we annotated and analyzed the conservedness of the PSPG (Plant Secondary Product GT) motif, which is a hallmark feature of plant UGTs [41]. From the 27 UGTs, we could clearly detect highly conserved PSPG motifs (Figure 2C). Thus, we identified 23 novel UGT candidates that may function in the catalysis of PPD or PPT during ginsenoside biosynthesis.

### 3.2. Three-Dimensional Structure Identified Various Conformations of Putative Ginsenoside Biosynthetic UGTs

**Figure 3:**
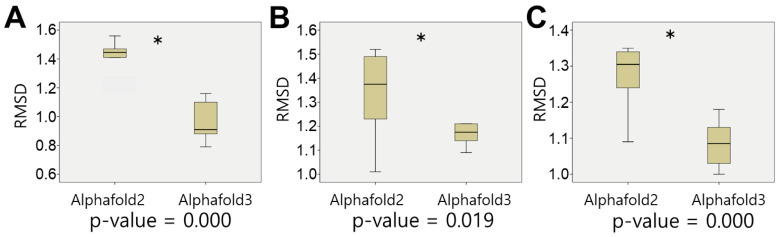
Identification of the appropriate model to predict the plant-type UGTs. Alphafold2 and Alphafold3 were applied 10 times to predict the 3D structures of three experimentally validated plant UGTs. The predicted structures were compared with their corresponding experimentally determined structures using RMSD as the metric. Statistical significance was identified with Student’s *t*-test as *p* < 0.001, which is indicated with the asterisk. (**A**) Model test on PDB:6JEM. (**B**) Model test on PDB:7Q3S. (**C**) Model test on PDB:8ITA.

Since the functional specificity of UGTs can be determined by only a few amino acid residues [23], we applied a 3D-structure-based approach to capture the structural nuances (Figure 1). First, we evaluated the accuracy of the prediction of the structure using AlphaFold2 and AlphaFold3 for three experimentally resolved plant UGTs (PDB IDs: 6JEM, 7Q3S, 8ITA). The RMSD values from pairwise structural alignments showed that AlphaFold3 produced more accurate models (Figure 3), and it was therefore used for all subsequent predictions. 

**Figure 4:**
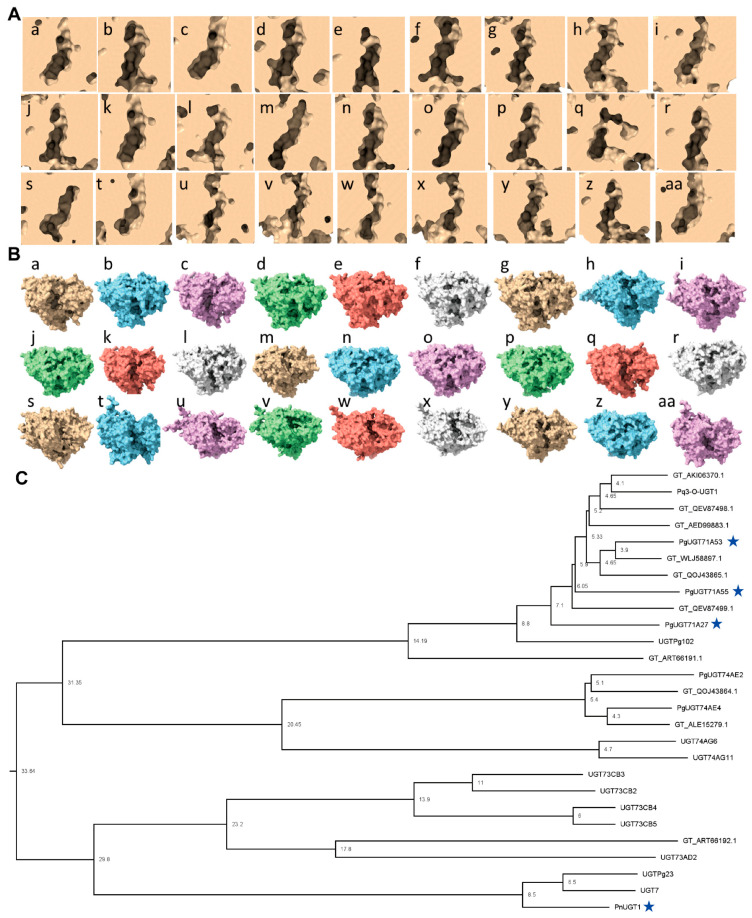
Three-dimensional structures of 27 UGTs identified in this study. Blue stars indicate the ginsenoside biosynthetic UGTs. All the structures were predicted with Alphafold3. Small characters of large characters denote each protein sequence in the following order: a. A0A0A6ZFR4.1 (PgUGT74AE2); b. A0A0A7HB61.1 (PgUGT71A27); c. A0A0D5ZDC8.1 (PgUGT74AE4); d. A0A0K0PVL0.1 (UGTPg102); e. A0A0K0PVM5.1 (PgUGT71A55); f. A0A068J840.1 (PgUGT71A53); g. AED99883.1 (glycosyltransferase); h. AFO63526.1 (PnUGT1); i. AKA44602.1 (UGTPg23); j. AKI06370.1 (glycosyltransferase); k. ALE15279.1 (UDP-glycosyltransferase 3GT1); l. ART66191.1 (uridine diphosphate glycosyltransferase1); m. ART66192.1 (uridine diphosphate glycosyltransferase2); n. QEV87497.1 (Pq3-O-UGT1); o. QEV87498.1 (UDP-glycosyltransferase); p. QEV87499.1 (UDP-glycosyltransferase); q. QOJ43864.1 (UDP-glycosyltransferase); r. QOJ43865.1 (UDP-glycosyltransferase); s. UMX47351.1 (UGT73AD2); t. UMX47352.1 (UGT74AG6); u. UMX47353.1 (UGT73CB3); v. UMX47354.1 (UGT73CB2); w. UMX47355.1 (UGT73CB4); x. UMX47356.1 (UGT73CB5); y. UNO37640.1 (UGT7); z. WLJ58897.1 (UDP-glycosyltransferase); aa. WPX61740.1 (UGT74AG11). (**A**) Representative view of potential active sites for the UGTs. The same position was calibrated through the PSPS motif sequence. (**B**) Representative view of the whole protein structures of the UGTs. (**C**) Structure-based phylogenetic tree.

We predicted the 3D structures of all 27 UGTs using AlphaFold3 and analyzed their putative active sites based on PSPG motif localization (Figure 4). Despite high sequence similarity (≥99%), the predicted active sites showed substantial conformational variation (Figure 4A and Figure S1). From a top-down perspective, the global protein architecture appeared to be similar (Figure 4B and Figure S1), suggesting that minor differences in terms of the primary sequence led to region-specific structural divergence. Structure-based phylogenetic analysis using the Dali server further supported these observations. Several predicted UGTs clustered closely with experimentally verified UGTs, while a distinct orphan clade emerged (Figure 4C). Interestingly, the orphan clade included PgUGT74AE2 and PgUGT74AE4, which are known to catalyze CK to F2 conversions, highlighting their potential involvement in broader ginsenoside metabolism. Building on these findings, we proceeded to investigate additional candidate UGTs that may catalyze the key reactions of interest in our study.

### 3.3. Functional Prediction of Ginsenoside Biosynthetic UGTs

**Figure 5:**
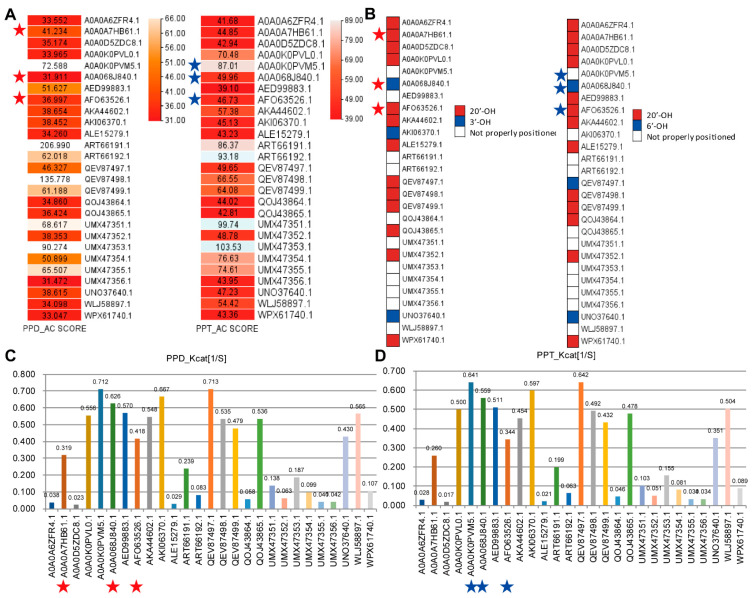
Functional annotation of the potential ginsenoside-catalyzing UGTs. Red stars indicate experimentally verified PPD-CK catalyzing enzymes, while blue stars indicate experimentally verified PPT-F1 catalyzing enzymes. (**A**) SwissDock prediction of intractability between UGTs and PPD or PPT. AC scores were visualized with a heatmap with color keys in a red gradient. (**B**) cb-dock2 prediction of intractability between PPD and PPT. The relative position of exposed carbon in PPD or PPT was represented with a heatmap representation. (**C**) MPEK prediction of K_cat_ between UGTs and PPD. (**D**) MPEK prediction of K_cat_ between UGTs and PPT.

Furthermore, we analyzed the potential functionality of the 27 UGTs (Figure 5). Sugar moieties can bind in C-3, C-6, and C-20 positions of the triterpenoid backbone of the ginsenosides [19]. Our reactions of interest comprise the catalytic reaction from PPD to CK, which contains the attachment of the glucose in C-20 position, and the catalytic reaction from PPT to F1, which also contains the attachment of the glucose in C-20 position (Figure 2A). Considering the detailed steps of enzymatic activity, the functional prediction of ginsenoside-catalyzing UGT should comprise both intractability with the ligand and the superposition of the ligand in UGT [42]. We applied two protein–ligand interaction prediction tools, SwissDock and cb-dock2 (Figure 5A,B). SwissDock targets a potential cavity site and then calculates the intractability [8,9]; thus, it ensures more precise predictability between the protein and the ligand. In our benchmarking analysis, SwissDock demonstrated an accuracy of 83.33% in predicting known interactions based on experimentally validated UGTs (Figure 5A). From the predicted interaction ability, the highest AC_score was set as the maximum allowable value to predict the intractability from 27 UGTs (Figure 5A). To analyze whether the predicted interactions would be suitable for experimental trials, we performed molecular dynamics prediction on all the protein–ligand combinations (Figure S2). Though few cases exceeded 2Å throughout the simulation periods, most of the combinations were stable (Figure S2). Thus, we concluded that our analyzed results would provide promising results in actual experiments (Figure S2).

Meanwhile, cb-dock2 detects cavities both through a protein structure-based and a ligand-preferred manner [10]. This means that it is impossible to use cb-dock2 to detect the relative intractability of all the protein–ligand pairs in the same superposition, yet cb-dock2’s predictability of the superposition of the ligand in interacting phase with UGT is better than that of SwissDock. Indeed, our benchmark result showed 83.33% predictability for cb-dock2 regarding the possible superpositions of the ligand inside the UGTs, while a predictability of only 66.66% was found for that of SwissDock (Figure 5B and Figure S3). Thus, we applied cb-dock2 to predict the superposition of the precursor interaction inside the UGT–ligand complex (Figure 5B).

Then, we predicted the catalytic activity using MPEK [12]. Although there are many other tools, such as Deepmolecules, MPEK contains plant-type catalytic reactions in its training sets [11,12]. Thus, we applied MPEK to predict the potential Kcat values, and they are visualized in Figure 5C. From these results, we identified AKA44602.1, which interacts with both PPD and PPT in a suitable position to attach a sugar moiety to catalyze into CK and F1, with comparable Kcat values of 0.548 and 0.454, respectively (Figure 5). QOJ43865.1 was revealed to be able to bind both PPD and PPT, yet only PPD was expected to bind in the right position to attach a sugar moiety in the 20’-OH position, with a comparable Kcat value of 0.536; thus, it was concluded that it can only catalyze PPD (Figure 5). QEV87498.1 and QEV87499.1 were predicted to only interact with PPT, exposing 20’-OH, with comparable Kcat values of 0.492 and 0.432, respectively; thus, it was concluded that it can only catalyze PPT (Figure 5).

To ensure the credibility of our simple pipeline, we performed molecular dynamics analysis for a longer period (Figure S4). In our analyzed UGTs, there are six UGT–ligand combinations which are experimentally shown to have catalytic activity: PgUGT71A27–PPD, PgUGT71A53–PPD, PnUGT1–PPD, PgUGT71A55–PPT, PgUGT71A53–PPT, and PnUGT1–PPT [22,23,43,44,45] (Figure S4A–F). We performed molecular dynamics simulations for 150 ns and analyzed the conformational stability of the system, the ligand, and an important residue in the UGT–triterpenoid interaction [40]. The five combinations, PgUGT71A27–PPD, PgUGT71A53–PPD, PnUGT1–PPD, PgUGT71A55–PPT, PgUGT71A53–PPT, and PnUGT1–PPT, reached a stable RMSD within 150 ns, and their local maxima did not exceed 0.4 nm (Figure S4A–E). In detail, PgUGT71A27 was moderately stable for 150 ns (Figure S4A). Residue 199 and the ligand were stable for 150 ns and were simulated to be under 0.2 nm, which ensured the stable interaction of PgUGT71A27 and the PPD molecule (Figure S4A). PgUGT71A53 was moderately stable, reaching RMSD of 0.35 nm within 150 ns (Figure S4B). Residue 199 and the ligand, meanwhile, were stable for 150 ns, with the local maximum staying near 0.2 nm, thus ensuring the stable interaction of PgUGT71A53 and PPD (Figure S4B). PnUGT1 was also moderately stable, reaching an RMSD of 0.4 nm within 150 ns (Figure S4C). Residue 203 was moderately stable, and the local maximum did not exceed 0.4 nm, while the ligand was highly stable, with a local maximum of 0.15 within 150 ns (Figure S4C). Thus, PnUGT1 was observed to interact with PPD in a stable state. PgUGT71A55 reached a local maximum after 100 ns, yet was moderately stable with a local maximal RMSD of 0.25 nm (Figure S4D). Residue 199 and the ligand were moderately stable with local maximal RMSD values of 0.25 nm, thus indicating that PgUGT71A55 stably interacts with PPT (Figure S4D). PgUGT71A53 was also moderately stable for 150 ns, with residue 199 and the ligand being stable for 150 ns with local maxima near 0.2 nm (Figure S4E). Thus, PgUGT71A53 was observed to interact with PPT (Figure S4E). However, PnUGT1’s interaction with PPT was found to be less stable, with a local maximum of 0.5 nm (Figure S4F). Thus, PnUGT1 was observed to have the potential for stable interactions with PPT, since the ligand RMSD was highly stable for 150 ns (Figure S4F). In short, molecular dynamics analysis indicated that the experimentally proven UGTs were successfully predicted through our pipeline with 83.33% accuracy (five of out six predicted), which ensures the credibility and reliability of our simple pipeline.

**Figure 6:**
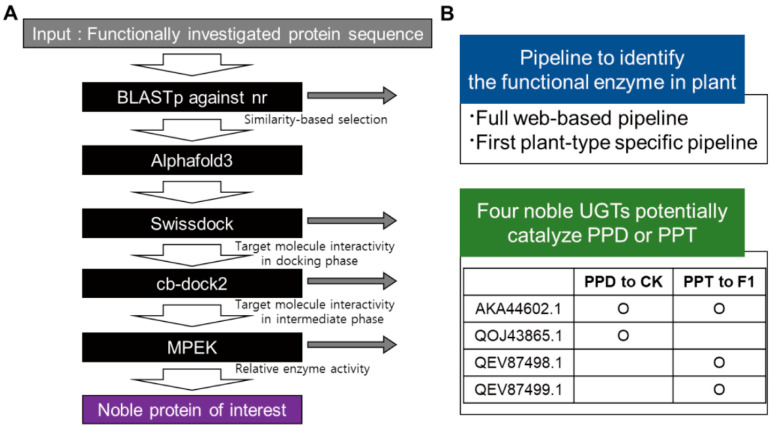
Summary of this study. (**A**) Schematic summary of structure-based pipeline for plant enzymes from this study. (**B**) Bullet summary of findings throughout this study.

Then, we performed molecular dynamics simulations on five novel UGT–ligand combinations to further validate their potential activity: UGTPg23–PPD, UGTPg23–PPT, QEV87498.1–PPT, QEV87499.1–PPT and QOJ43865.1–PPD (Figure S4G–J). Among these, QEV87498.1–PPT was found to induce an energy blow-up. Thus, a simulation could not be performed, which indicates that this combination cannot be functional. The other four combinations were analyzed for their molecular dynamics for 150 ns (Figure S4G–J). UGTPg23 was moderately stable in its interactions with both PPD and PPT, for which the local maxima did not exceed 0.4 nm for 150 ns (Figure S4G,H). PPT was found to be more stable when interacting with UGTPg23 than PPD because the local maximum of the ligand RMSD was higher in UGTPg23–PPD than in UGTPg23–PPT (Figure S4G,H). QEV87499.1 was moderately stable when interacting with PPT for 150 ns (Figure S4I). Since residue 199 and PPT were stable with a local maximal RMSD lower than 0.2 nm, QEV87499.1 was expected to stably interact with PPT (Figure S4I). QOJ43865.1 was moderately stable for 150 ns when interacting with PPD, with residue 199 and PPD being stable for 150 ns (Figure S4J). In summary, our pipeline was able to predict the functionality of both experimentally proven UGTs and noble UGTs at a rate of more than 80%, which will be further assessed in the future (Figure 6).

## 4. Discussion

We present a structure-guided computational pipeline tailored to the functional prediction of plant enzymes, exemplified by the identification of ginsenoside biosynthetic UGTs (Figure 6). The pilot application of our pipeline identified four candidate UGTs potentially involved in PPD-to-CK or PPT-to-F1 conversion (Figure 5). Importantly, all of the analytical tools used in this pipeline are user-friendly and available as web-based platforms, making them accessible to plant researchers unfamiliar with Linux environments.

Despite its utility, the pipeline is limited by the paucity of experimentally validated plant protein data, which is a common limitation in plant structural bioinformatics. Nevertheless, the framework is broadly applicable to several enzyme classes, including hydrolases, transferases, oxidoreductases, lyases, isomerases, ligases, and translocases (Figure 6). The pipeline’s prediction accuracy may vary depending on the representation of enzyme classes in the PDB; for example, hydrolases and transferases are better represented than translocases or extracellular enzymes, which may affect the performance of the pipeline for proteins localized outside the cytoplasm.

Some of the UGT nominees were able to interact with PPD or PPT in the right superposition, yet the Kcat value was very low, which made us reject them as putative ginsenoside processing UGTs (Figure 5). For instance, A0A0A6ZFR4.1, which encodes PgUGT74AE2, was able to interact with PPD, exposing 20’-OH, yet the Kcat value was only expected to be 0.038 [22] (Figure 5). We had the same problem in the case of A0A0D5ZDC8.1, which encodes UGT45, where the predicted Kcat value was 0.023 [46] (Figure 5). These two enzymes were predicted to be able to interact with PPT, as well as with the sugar moiety attachable composition, yet low Kcat values of 0.028 and 0.017 were found (Figure 5). Both UGTs were shown to catalyze PPD into Rh2; thus, our pipeline correctly predicted the functionality [22,46]. Similar cases were also found for unknown enzymes such as ALE15279.1, UMX47352.1, and WPX61740.1 (Figure 5). Thus, these enzymes might provide other target UGTs that may be able to mediate PPD into other compounds such as Rh2 (Figure 2A). QEV87497.1 was a slightly different case, as it has a high chance of being able to bind with PPD, and it had a superior Kcat value (Figure 5). However, the AC_score of QEV87497.1 was 46.33, which is greater than the highest AC_score predicted from the experimentally proven UGTs, which is 41.23 for A0A0HB61.1 (Figure 5). Thus, this enzyme requires further validation through re-design or testing.

Of the four predicted UGTs with potential catalytic activity in PPD to CK or PPT to F1, only one UGT was identified from *P. ginseng* (Figure 5). Recently, the telomere-to-telomere (T2T)-level genome of *P. ginseng* was published [47]. Thus, we confirmed the potential proteins at the genome level through BLASTp, and we identified that AKA44602.1 was pg_9002689 in the T2T-level genome. Moreover, AKA44602.1 was revealed to be a highly similar gene to UNO37640.1. This result further suggests the need to confirm the existence of the potential genes through our pipeline in the actual genome structure.

The predicted structures of our pipeline indicate that the PSPG motif and precursor have a binding cavity to reside in nearby (Figure 4A,B). Unexpectedly, our result showed a huge variety in the morphology of the precursor binding cavity (Figure 4A). The reason for this variety might be caused by different target ligands other than ginsenosides, considering the vast variety of the triterpenoid saponin molecules [48]. Another possibility could be raised regarding the genomic duplication, which might be true for the relationship between AKA44602.1 and UNO37640.1. Our results indicate that these two proteins are the closest homologs in a sequence-based and structure-based manner (Figure 2B and Figure 4C). AKA44602.1 was not detected in the T2T genome annotation, suggesting either extremely low expression levels or an atypical gene structure. Thus, these considerations should be further assessed to narrow down the targets that could be applied successfully in our pipeline.

## Figures and Tables

**Table 1:** Target biosynthetic process and recognized enzymes.

Precursor	Product	Enzyme (UGT)	UniProtKB ID
Protopanaxadiol	Ginsenoside CK	PgUGT71A27	A0A0A7HB61.1
		PgUGT71A53	A0A068J840.1
		PnUGT1	AFO63526.1
Protopanaxatriol	Ginsenoside F1	PgUGT71A53	A0A068J840.1
		PgUGT71A55	A0A0K0PVM5.1
		PnUGT1	AFO63526.1

## Data Availability

The original contributions presented in this study are included in the article/Supplementary Material. Further inquiries can be directed to the corresponding author(s).

## Supplementary Materials

The following supporting information can be downloaded at: https://www.mdpi.com/article/10.3390/biotech14030073/s1, Figure S1: Superimposed image of the structures of all the analyzed UGTs.; Figure S2: Molecular dynamics analysis was performed for all the protein-ligand combination.; Figure S3: Actual example of UGT-ligand interaction.; Figure S4: Long time molecular dynamics analysis on the ten representative cases analyzed in this study.
