# Supplementary material for: Structure-Based Pipeline for Plant Enzymes: Pilot Study Identifying Novel Ginsenoside Biosynthetic UGTs
Source: BioTech (Basel). 2025 Sep 12;14(3):73. doi: 10.3390/biotech14030073 (PMC12452368; doi:10.3390/biotech14030073)
Supplement: Supplementary file 1 [file biotech-14-00073-s001.zip › biotech-3672720-supplementary.pdf]

Supplementary Material

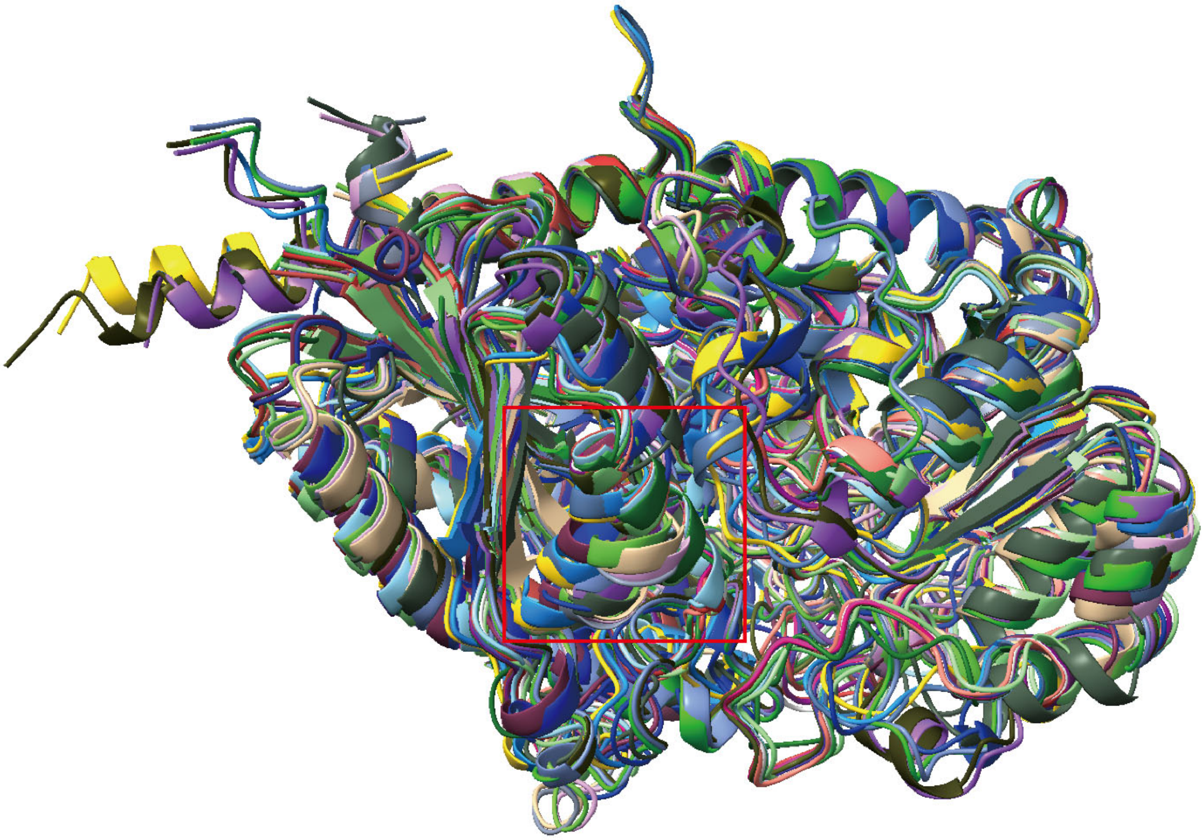

Figure S1. Superimposed image of the structures of all the analyzed UGTs. Red boxed region indicates the sugar-moiety-interacting region diversity.

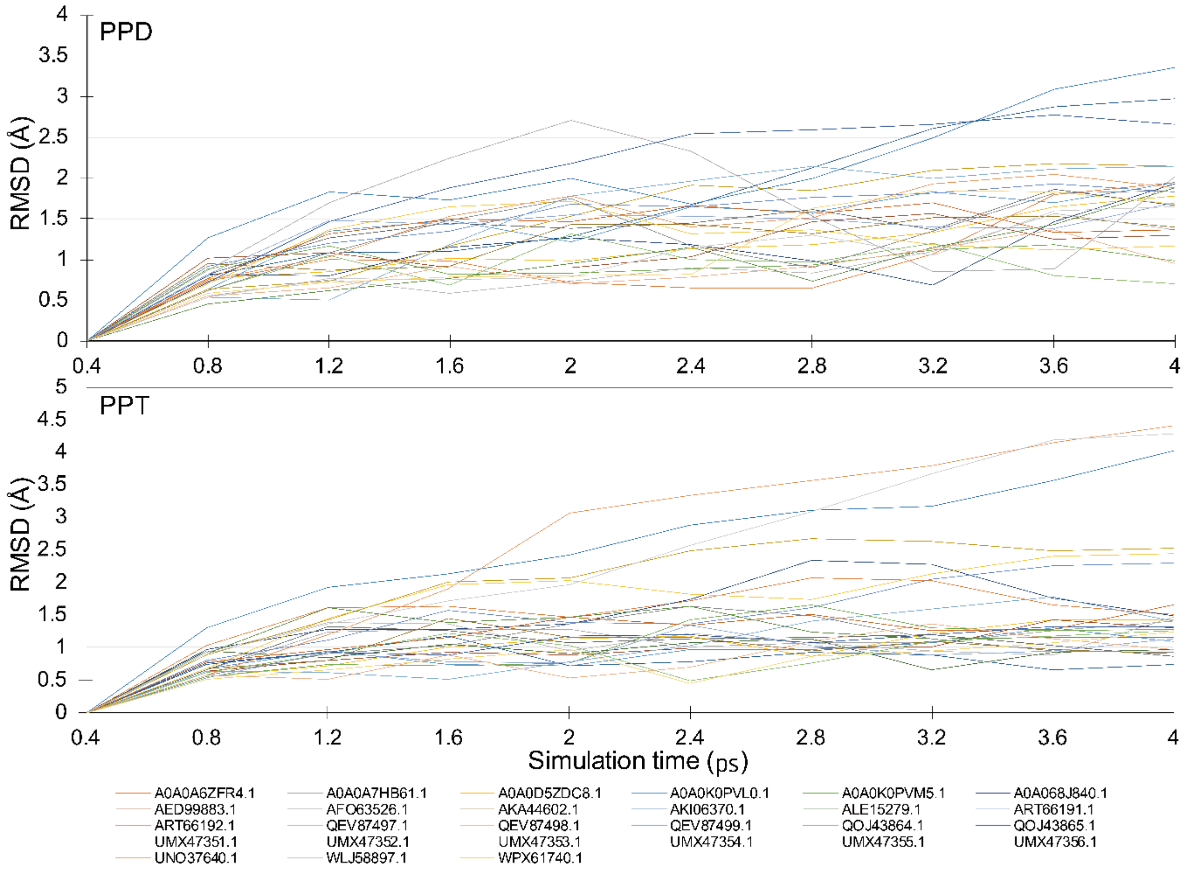

Figure S2. Molecular dynamics analysis was performed for all the protein-ligand combination. RMSD of the ligands were visualized for four picoseconds. Proteins were annotated with each color and ligand type was separated by different graph. Two graphs share the same color scheme.

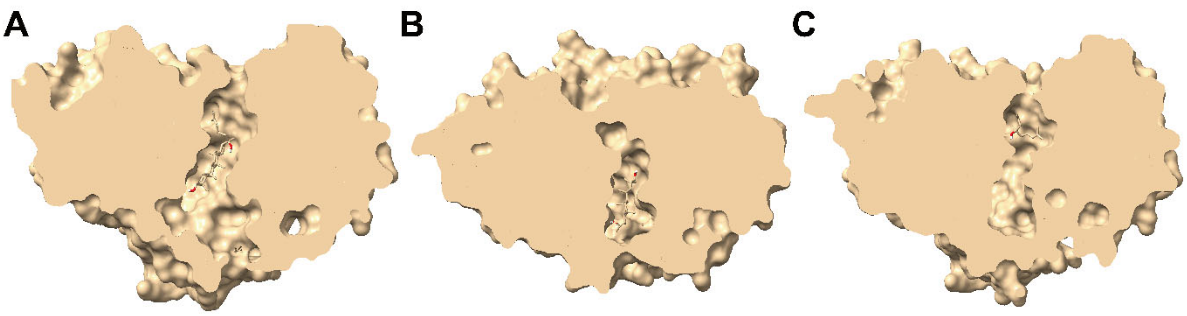

Figure S3. Actual example of UGT-ligand interaction. **A.** PgUGT71A27 (A0A0A7HB61.1) which interacts with PPD by exposing 20′-OH to UDP-sugar. This interacting position is able to catalyze PPD into CK. **B.** PgUGT71A53 (A0A068J840.1) which interacts PPD by exposing 3′-OH to UDP-sugar. This position is impossible to mediate PPD into CK. **C.** PgUGT71A55 (A0A0K0PVM5.1) which interacts PPD with abnormal position.

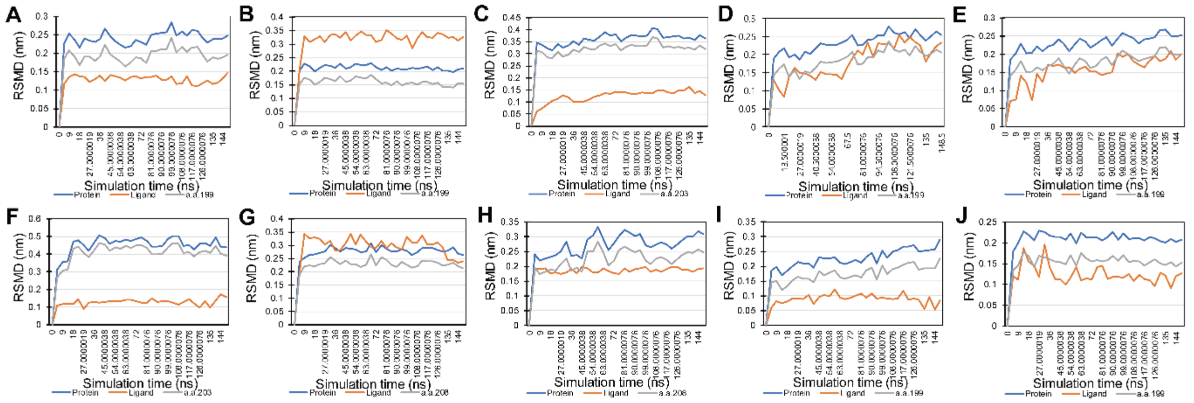

Figure S4. Long time molecular dynamics analysis on the ten representative cases analyzed in this study. Blue line indicates whole protein RMSD, orange line indicates ligand (PPD or PPT) and grey line indicates the key amino acid for interaction between UGT and ligand molecule. Each key amino acid was determined by literature study and sequence alignment. All the molecular dynamics were analyzed for 150 ns. **(A-F)** Six UGT-ligand combinations of known catalytic activity. **A.** Molecular dynamics analysis on PgUGT71A27-PPD pair. **B.** Molecular dynamics analysis on PgUGT71A53-PPD pair. **C.** Molecular dynamics analysis on PnUGT1-PPD pair. **D.** Molecular dynamics analysis on PgUGT71A55-PPT pair. **E.** Molecular dynamics analysis on PgUGT71A53-PPT pair. **F.** Molecular dynamics analysis on PnUGT1-PPT pair. **(G-J)** Four UGT-ligand combinations to have potentially catalytic activity through this study. **G.** Molecular dynamics analysis on UGTPg23-PPD pair. **H.** Molecular dynamics analysis on UGTPg23-PPT pair. **I.** Molecular dynamics analysis on QEV87499.1-PPT pair. **J.** Molecular dynamics analysis on QOJ43865.1-PPD pair.
